# Component Analysis and Anti-Colorectal Cancer Mechanism via AKT/mTOR Signalling Pathway of *Sanghuangporus vaninii* Extracts

**DOI:** 10.3390/molecules27041153

**Published:** 2022-02-09

**Authors:** Shanshan Guo, Wenwen Duan, Yaxin Wang, Liangmian Chen, Chenchen Yang, Xuezhu Gu, Qinghai Xue, Raorao Li, Zhijie Zhang

**Affiliations:** 1 Institute of Chinese Materia Medica, China Academy of Chinese Medical Sciences, Beijing 100700, China; ssguo@icmm.ac.cn (S.G.); 18811381928@163.com (W.D.); wyx_18434376603@163.com (Y.W.); lmchen@icmm.ac.cn (L.C.); chenchenyang1994@163.com (C.Y.); xzgu@icmm.ac.cn (X.G.); 2 School of Pharmaceutical Sciences, Zhengzhou University, Zhengzhou 450001, China; 3 The TCM Clinic of China Academy, China Academy of Chinese Medical Sciences, Beijing 100700, China; haihai0730@163.com

**Keywords:** *Sanghuangporus vaninii* (Ljub.) L.W. Zhou & Y.C. Dai (SV) extracts, component analysis, colon cancer, AKT/mTOR

## Abstract

*Sanghuangporus vaninii* (Ljub.) L.W. Zhou & Y.C. Dai (SV) is a major cultivar of Sanghuang, which is well known as an excellent anti-tumour drug and reaches the mainstream market in China. Water, 60% ethanol and 95% ethanol were used to extract the drug, and three kinds of polar extracts were obtained separately. Compared with water extracts and 95% ethanol extracts, the 60% ethanol extract had the highest flavonoid content, and its polysaccharide content was greater than that in the 95% ethanol extract and lower than that in the water extract. Its essential components were phenolics whose majority were phenolic acids, flavonoids and phenylpropanoids. This extract has better inhibition effects on the proliferation of SW480 human colon cancer cells, inducing cell apoptosis and blocking G2/M period cells. It can significantly inhibit gene expression and reduce the activation of the AKT/mTOR signalling pathway. The anti-cancer activity of the 60% ethanol extract is satisfactory and may be a result of the combined effects of polysaccharides and flavonoids. The data suggest that the 60% ethanol extract can be used as an adjuvant for chemotherapy and as a potential anti-cancer agent with broad development prospects.

## 1. Introduction

Colon cancer is the third most common cancer in the world and ranks fourth in cancer mortality [1,2]. The mammalian target of rapamycin (mTOR) is a 289 kDa serine/threonine kinase which belongs to the Akt phosphorylated downstream effector family. It can control cell growth and regulate cancer cell survival and proliferation [3,4]. The Akt/mTOR signalling pathway plays an important role in the survival and growth of cancer cells, such as colon cancer cells [5]. Phytomedicine has become the focus of colon cancer research due to its mild medicinal effect and few side effects [6].

Sanghuang is a large perennial medicinal fungus that parasitizes the woody substrate of Morus plants. Sanghuang has strong inhibitory effects on tumours, and it is widely used in Japan and South Korea [7]. It was first recorded in *Shen Nong’s Materia Medica* and was considered to be a high-grade herb. Sanghuang is stated to have an “elimination of toxicant” (“pai du qi” in Chinese) function in the *New Compendium of Materia Medica* (Xin Xiu Ben Cao) and *Chinese Compendium of Materia Medica* (Ben Cao Gang Mu) [8,9]. Pharmacological studies have revealed that Sanghuang possesses a variety of biological activities, including anti-cancer, immunoregulation, anti-diabetes, antioxidation and anti-inflammation activities. Many kinds of compounds, such as polysaccharides, flavones, triterpenes, aromatic acids and amino acids, have been reported in *Sanghuang*, and the largest proportion of compounds in the drug are polysaccharides [10]. According to the research of the Japanese scholar Tetsuro Ikekawa, the wild fruit body extract of *Sanghuang* has a 96.7% inhibitory rate on mouse sarcoma S180, with little toxicity towards normal cells [11]. Many studies have also shown that Sanghuang polysaccharides have significant anti-cancer activity [12,13]. Ge Li et al. reported that polysaccharides have an inhibitory effect on the proliferation and colony formation of SW480 human colon cancer cells, and the effect is associated with decreased Bcl-2 expression, increased release of cytochrome *c* and reduced cyclin B1 expression [14]. The study also showed that Sanghuang polyphenols have a significant anti-cancer effect, as the polyphenols had cytotoxic activities against various cancer cells [15]. In the treatment of medullary thyroid cancer, researchers found that citric acid did improve symptoms caused by cancer [16]. Takuji Tanaka et al. [17] reported that PA can protect against the development of epithelial malignancy in different tissues and cardiovascular diseases. The mechanism of action mostly involves antioxidant activity, such as inhibiting free radical generation and scavenging and upregulating antioxidant enzymes. By performing a caffeic acid-mediated facile synthesis of silver nanoparticles, the study demonstrated that PA could enhance anti-cancer activity [18].

*Sanghuangporus vaninii* is responsible for the largest amount of Sanghuang in markets. However, many studies have mainly focused on the extraction, isolation and component analysis of *S. vaninii*, and there are few studies regarding its bioactivity [7]. Qiong Guo et al. found that *S. vaninii* exhibits a strong capacity for free radical scavenging, can effectively alleviate cellular oxidative stress and provides therapeutic effects on gout [19]. Xilin Wan et al. reported that polysaccharide from *S. vaninii* have anti-tumour functions through the activation of the p53 signalling pathway in breast cancer MCF-7 cells. The anti-colon mechanism of *S. vaninii* has been reported scarcely. In this article, three kinds of extracts were extracted by different polar solvents (water, 60% ethanol and 95% ethanol). The effects on the proliferation, cell cycle and apoptosis of SW480 colon cancer cells in vitro were used as indicators. qRT-PCR and Western blotting were used to explore the effects of the extracts on the AKT/mTOR pathway and preliminarily explain their mechanism of inhibiting human colon cancer cells.

## 2. Results

### 2.1. Total Polysaccharide and Flavonoids Contents

The total polysaccharide and flavonoid contents of SVW (water extract of S. vaninii), SVE60 (60% ethanolic extract of *S. vaninii*) and SVE95 (95% ethanolic extract of *S. vaninii*) were 0.626, 0.342 and 0.337 mg/mg and 0.0481, 0.5574 and 0.4843 mg/mg, respectively. The polysaccharide content of SVW was the highest, and that of flavonoids was the lowest. The polysaccharide content of SVE60 was slightly higher than that of SVE95, and the total flavonoid content was the highest in SVE60, which was 11.58 times as high as that in SVW and 1.15 times as that in SVE95.

### 2.2. UPLC/Q-TOF-MS Analysis

**Figure 1:**
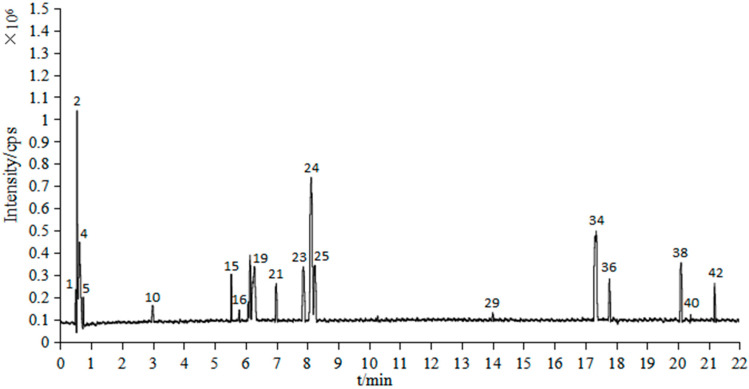
Total ion chromatogram of UPLC-Q-TOF-MS (positive mode) of SVE60.

**Figure 2:**
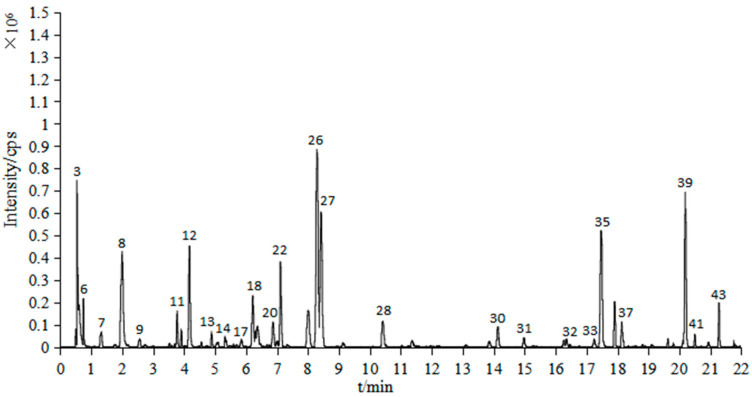
Total ion chromatogram of UPLC-Q-TOF-MS (negative mode) of SVE60.

In the 60% ethanol extract of *S. vaninii*, 43 compounds were obtained, including 35 compounds tentatively identified by comparison of absorption spectra with the online database ChemSpider, and reported values in the literature together with the remaining 8 were not identified by either absorption or mass spectra [20,21,22,23,24,25,26,27,28,29,30,31,32,33,34,35,36,37,38,39] (Table 1). The results showed that phenolics were the essential component of SVE60, including phenolic acids, flavonoids and phenylpropanoids (Figure 1 and Figure 2).

### 2.3. The Result of CCK8 Cell Proliferation

Cancer cells are unmanageable, chronic and have obvious characteristics, including a high proliferation ability and an ability to rapidly migrate [40,41]. To demonstrate whether the extracts of *S. vaninii* have an inhibitory effect on SW480 human colon cell proliferation in a dose-dependent manner, the experiments involved three groups. The SVW group (the control group, group 1), SVE60 (group 2) and SVE95 (group 3) were each treated with the same dosages (0, 1.2, 3.7, 11.1, 33.3, 100 and 300 μg/mL) and effective times (72 h).

**Figure 3:**
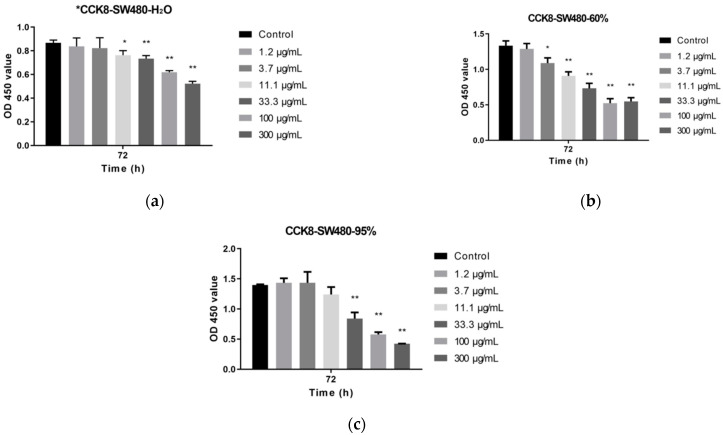
Effect of *S. vaninii* extracts on the proliferation of SW480 cells (* *p* < 0.05, ** *p* < 0.01) (**a**) SVW, (**b**) SVE60 and (**c**) SVE95.

Compared with that of the control group, the OD values of the three extracts gradually decreased with increasing dose levels after administration for 72 h. There were significant differences in the SVW group (300, 100, 33.3 μg/mL dose groups) (Figure 3a), SVE60 group (300, 100, 33.3, 11.1 and 3.7 μg/mL dose groups) (Figure 3b) and SVE95 group (300, 100 and 33.3 μg/mL dose groups) (Figure 3c). The three extracts could significantly reduce the cell survival rate with increased dosage, especially the 300, 100 and 33.3 μg/mL dose groups (*p* < 0.01).

Compared with the SVW and SVE95, SVE60 showed an inhibitory effect on the proliferation of colon cancer cells SW480 at a lower concentration (3.7 μg/mL), with a significant difference (*p* < 0.05). The IC_50_ values of SVW, SVE60 and SVE95 were 209.5, 7.91 and 27.14 μg/mL. The effect of two ethanol extracts was more obvious than that of the water extract, particularly the 60% ethanol extract of *S. vaninii,* whose IC_50_ was only one third of 95% ethanol extract and one twenty-sixth of water extract, approximately.

### 2.4. Induction of Apoptosis by the Ethanol Extracts of S. vaninii

Apoptosis is the key process in inhibiting the proliferation of cancer cells [42]. Annexin-V can specially bind with the early stage of apoptosis and provide FITC labels [43]. Propidine iodide (PI) is a nucleic acid dye that can penetrate the cell membrane in the middle and advanced stages of apoptosis to incarnadine nuclei. Therefore, Annexin-V and PI are used simultaneously in experiments to identify and differentiate early, middle and advanced apoptosis and necrotic cells [44]. This procedure was adopted in our research, and flow cytometry was applied to detect the apoptosis rates.

**Figure 4:**
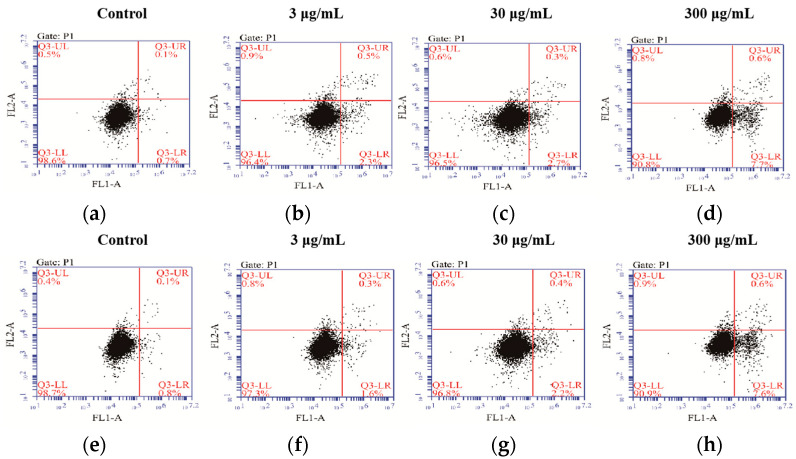
Effect of *S. vaninii* extracts on the apoptotisis of SW480 cells. Scheme 60. Group: (**a**) control group, (**b**) 3 μg/mL dose group, (**c**) 30 μg/mL dose group and (**d**) 30 μg/mL dose group. Scheme 95. Group: (**e**) control group, (**f**) 3 μg/mL dose group, (**g**) 30 μg/mL dose group and (**h**) 30 μg/mL dose group.

There were two groups in the experiment, the SVE60 (Figure 4a–d) and SVE95 group (Figure 4e–h), which were divided into four dose groups (the control group, 3, 30 and 300 μg/mL dose groups). Then, the cells were stained with Annexin V and PI.

Compared with the control group, the numbers of late apoptotic cells and early apoptotic cells were greater with multiplied dosages (10 times) of SVE60 and SVE95 (Figure 3). The results showed that the ethanol extracts of *S. vaninii* induced the SW480 apoptosis in a certain dose-dependent manner, and the effect of SVE60 was slightly superior to that of SVE95 (Table 2).

### 2.5. Effect of SVE60 on Cell Cycle Progression

The cell cycle is an important factor in cell proliferation, and its process begins with one cell division and ends when the next cell division is finished [45]. The cell cycle process was analysed via flow cytometry, and the study used SVE60 as the drug. The control group, and 3, 30 and 300 μg/mL dose groups were used. Compared with that of the control group, the proportion of cells in the G0/G1 phase was decreased by 6.08%, 1.82% and 9.53% at 3, 30 and 300 μg/mL, and those in G2/M phase increased by 7.75%, 0.61% and 9.36%, respectively. The proportion of cells in S phase was increased by 1.21% and 0.16% at 30 and 300 μg/mL, respectively, but decreased by 1.68% at 3 μg/mL.

**Figure 5:**
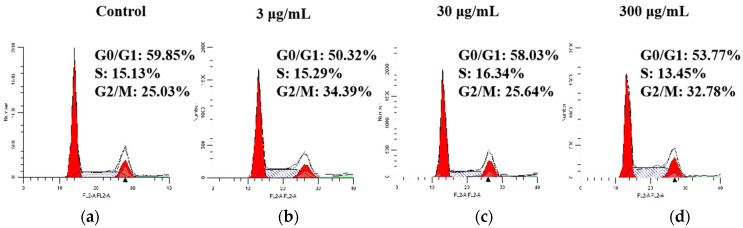
Effect of SVE60 on cell cycle progression: (**a**) control group, (**b**) 3 μg/mL dose group, (**c**) 30 μg/mL dose group and (**d**) 30 μg/mL dose group.

The proportion of cells in the S or G2/M phase was influenced by different drug concentrations. The 3 μg/mL dose decreased the proportion of cells in the S phase and increased the proportion of cells in the G2/M phase. The 30 μg/mL dose group induced apoptosis of cells derived from the G0/G1 phase. The results (Figure 5) showed that SVE60 was able to inhibit cancer cell proliferation by causing cell arrest in different phases.

### 2.6. The Result of qRT-PCR

**Figure 6:**
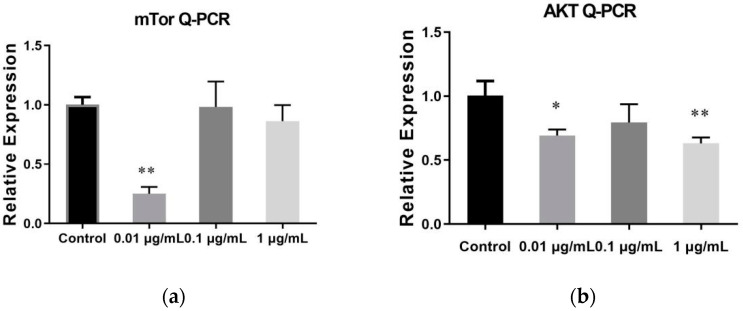
Effect of SVE60 on mTOR and AKT gene expression (**a**) mTOR. (**b**) AKT (* *p* < 0.05, ** *p* < 0.01).

The study observed the influence of SVE on the mTOR and AKT gene expression in the mTOR signalling pathway (Figure 6). There were significant differences between the SVE60 0.01 μg/mL dose group, which had an obvious inhibitory effect on mTOR gene expression, and the control group. Three dose groups showed a decreasing trend in AKT gene expression. The data demonstrated that SVE60 had a significant inhibitory effect on the expression of key genes in the mTOR signalling pathway.

### 2.7. The Result of SVE60 on the Protein Expression via mTOR Signalling Pathway

**Figure 7:**
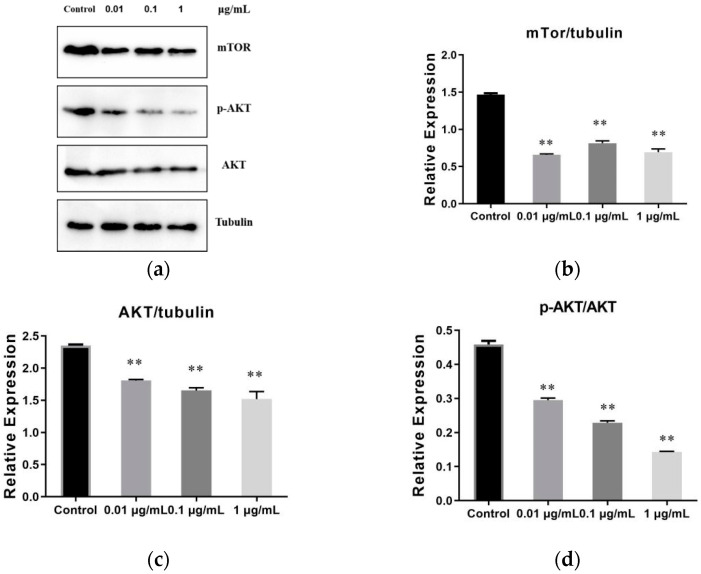
Effect of SVE60 on the protein expression via mTOR, AKT and p-AKT (**a**) Western blotting, (**b**) mTOR, (**c**) AKT, (**d**) p-AKT. (** *p* < 0.01).

Tubulin was used as an internal reference protein to observe the influence of SVE60 on the mTOR, AKT and p-AKT proteins in the mTOR signalling pathway (Figure 7). The results showed that the three dose groups significantly decreased the expression of mTOR, AKT and p-AKT proteins. SVE60 exhibited an obvious dose-dependent relationship with the expression of AKT and p-AKT proteins. This result indicated that the inhibitory effect of SVE60 on the proliferation of SW480 cells was related to the activation of mTOR signalling.

## 3. Materials and Methods

### 3.1. Materials

The samples used in this research were collected from Jilin Province and identified as *Sanghuangporus vaninii* (Liub.) L.W. Zhou & Y.C. Dai by Professor Dai Yucheng from Beijing Forestry University. The human colon cancer cell line SW480 was purchased from Sai Bai Kang (Shanghai, China). Primary antibodies including p-AKT (ab38449, 1 mg/mL), AKT (ab179643, 1 mg/mL) and mTOR (ab2732, 1 mg/mL) were purchased from Abcam (Cambridge, Britain). Foetal bovine serum (16000-044) was obtained from Gibco (Grand Island, NY, USA). RIPA Protein Lysis Buffer was purchased from Xin Sheng Yuan (Beijing, China). The BCA ELISA Kit (CW0014S) was purchased from Kang Wei Shi Ji (Shenzhen, China). A dual-beam UV–Vis spectrophotometer (CW0014S) was purchased from Agilent Technologies (Santa Clara, CA, USA). The flow cytometer (C6) was purchased from BD (East Rutherford, NJ, USA). The ELISA (Spectra Max M5) was purchased from Molecular Devices, (San Jose, CA, USA). The cell incubator (311) was purchased from Thermo (Waltham, MA, USA).

### 3.2. Preparation of the Phellinus Linteus Extracts

The powder (20 g, approximately) of *S. vaninii* was extracted with 400 mL of pure water for 1 h in a water bath. The extract was centrifuged at 5000 r/min for 5 min. The supernatant was concentrated under vacuum and then lyophilized using a vacuum freeze dryer. The water extract of *S. vaninii* (SVW) was stored for further use. The 60% and 95% ethanolic extracts (SVE60 and SVE95) were prepared with the same method as SVW, and the supernatants were evaporated in vacuum until dry.

### 3.3. Total Polysaccharide Content

The polysaccharide content was analysed by the glucose calibration, which was determined using the anthrone-sulfuric acid method [46]. SVW, SVE60 and SVE95 were diluted with distilled water (*w*/*v*, 1:5), and 1.0 mL of aqueous solution was used for measurement. The solution was added to 2.0 mL with distilled water, and 6.0 mL of 0.2% anthrone-sulfuric acid was added slowly and heated at 100 °C for 15 min, and absorbance measurements were recorded at 625 nm.

### 3.4. Total Flavonoids Content

The total flavonoid content was measured by sodium nitrite–aluminium nitrate colorimetry through UV spectroscopy using rutin as a reference substance at a concentration of 0.20 mg/mL [47]. The control solutions 1.0, 2.0, 3.0, 4.0, 5.0 and 6.0 mL were added, and distilled water was added to 6.0 mL. Then, 1.0 mL of 5% sodium nitrite was added to the solutions and incubated for 6 min separately. Next, 1.0 mL of 10% aluminium nitrate was added and reacted for 6 min. Finally, 10.0 mL of NaOH was added, distilled water was added to increase the liquid level, and the solution was shaken and developed for 15 min. The test solution and water were the blank controls, and a 500 nm wavelength was used. To formulate the standard curve, the concentration of rutin (C) was used as the X-axis and the Y-axis was the absorbance (A). The extracts were dissolved in methanol (*w*/*v*, 1:1), and the filtrates were measured in the same way.

### 3.5. UPLC-Q-TOF-MS Analytical Conditions

The UPLC-QTOF-MS/MS analysis was performed on a Waters Acquity UPLC/Xevo G2-S Q/TOF system (Waters Corporation, Milford, MA, USA) coupled with an electrospray ionization (ESI) source operating in both the positive and negative ionization modes. Chromatographic separation was performed on an Acquity UPLC BEH C_18_ column (2.1 × 100 mm, 1.7 μm) at 35 °C and 280 nm, and the flow rate was 0.4 mL/min. The mobile phases were a 0.1% formic acid aqueous solution (A) and acetonitrile (B) with a gradient elution program of 10–15% B at 0–2 min, 15–23% B at 2–4 min, 23% B at 4–8 min, 23–30% B at 8–11 min, 30% B at 11–14 min, 30–40% B at 14–20 min, 40–100% B at 20–23 min, 100–100% B at 23–25 min and 10% B at 25.01–28 min. The MS conditions were set as follows: a total ion scanning range of m/z 100–1200, nebulizer nitrogen gas flow rate 500 L/h, capillary voltage of 1.8 KV, cone voltage of 40 V, ionization temperature of 120 °C and desolvation temperature of 500 °C. The samples were collected by the MSE Centroid model, and the time taken was 25 min with a scan range of m/z 100–1500 Da and an interval time of 0.1 s. The ramp trap collision energy was set at 25–45 eV for the high-energy function and 6 eV for the low-energy function. Leucine-enkephalin was used as an external reference and was infused at a constant flow of 10 mL/min. All MS data were acquired by MassLynx 4.1 software. The SVE60 was dissolved in 1 mL of methanol with ultrasonication (53 kHz) for 30 min and then filtered through a 0.22 μm membrane filter for UPLC-MS analysis.

### 3.6. Cell Culture

The human colon cancer cell line SW480 was cultured in 1640 culture fluid with 10% foetal bovine serum. The cells displayed monolayer growth in a 37 °C atmosphere of 95% relative humidity and 5% CO_2_. Cells at logarithmic growth phase were used in the experiment.

### 3.7. CCK-8 Assay

The human colon cancer cell line SW480 was inoculated in 96-well culture plates at a density of 1 × 10^5^ per mL per well. The clear supernatant of SW480 cells that would be used for detection was poured out, and the cells were washed twice with PBS. A total of 100 μL of fresh L-15 complete medium and 10 μL of CCK-8 reagent was used per well. Then, the plates were placed in an incubator for 3 h, and the optical density (OD) was detected at 450 nm using a microplate reader.

### 3.8. Flow Cytometry Cell Apoptosis Analysis

The cells were pre-treated before the formal experiment. The colon cell SW480 was digested with trypsin. The adherent cells were washed with PBS three times and collected into centrifuge tubes. The supernatant was discarded after centrifugation at 2600 r/min for 5 min. The binding buffer was diluted 10 times with deionized water. The single-cell suspension was prepared by mixing the primed cells with 250 μL of diluted binding buffer. Then, the 50 μL single-cell suspension was removed from the 1.5 mL centrifuge tube, and the cells were stained with 2.5 μL of Annexin V-FITC and 5 μL of propidium iodide (PI) for 15 min at ambient temperature. Apoptosis was detected through flow cytometric analysis.

### 3.9. Cell Cycle Analysis

The colon cell line SW480 was prepared as a single-cell suspension through the same method as described in 2.8. The pre-treated cells were put into a tube, strained with 1 mL of DNA staining solution and 10 μL of permeabilization solution, and were mixed by vortex oscillation for 5–10 s. Then, the cells were incubated for 30 min at room temperature and were subjected to flow cytometry.

### 3.10. Quantitative RT-PCR

Total RNA was extracted from the cells with TRIzon reagent. The effect on the pathway mediated by mTOR and AKT was determined using the qRT-PCR method using a variety of extracts doses, such as blank, 0.01 μg/mL, 0.1 μg/mL and 1 μg/mL. A quantitative PCR instrument (Bio-Rad CFX96, Hercules, CA, USA) was used for the PCR, and the following primer sequences were used for the qRT-PCR (Table 3).

### 3.11. Western Blot

The expression of mTOR, p-AKT and AKT protein was detected after the extracts reacted with SW480 cells for 48 h and the antibodies, including mTOR, p-AKT and AKT, were diluted at a ratio of 1:1000. SW480 cells were washed with PBS three times, RIPA protein lysis buffer was added, the mixture rested for 30 min, and the cells were centrifuged at 12,000 rpm at 4 °C.

The supernatant liquid was obtained and the concentration of protein in the samples was detected using BCA test kits. Total protein was obtained and rinsed with TBST after sample filling, electrophoresis, transmembrane and closure was performed. Extracts with different concentrations, such as 0, 0.01, 0.1 and 1 μg/mL, were added, and then the mixture was stored overnight at 4 °C. Then, the mixture was rinsed with TBST again. After exposure and imaging, tubulin was used as a reference protein (at 1:1000 dilution) and Image Quant TL software was used to analyse the grey value of every belts.

### 3.12. Statistical Analysis

All quantitative data are presented as the means ± standard deviation (SD). The data were analysed by *t*-tests using GraphPad Prism 9.0 software. Differences were considered statistically significant when *p* < 0.05.

## 4. Conclusions and Discussion

Sanghuang has long been known to possess anti-cancer abilities. *S. vaninii* is its most widespread cultivar and provides rich resources for the application of this plant. To explore the medical usage of this drug, the relationship between the active compounds and anti-colon cancer was studied in this experiment.

Phenolic compounds and polysaccharides are the two main bioactive chemical groups with medicinal properties of Sanghuang [48]. In the study of Yang, the proximate compositions and microelements of Sanghuang were determined, including the contents of total phenolics, flavonoids and polysaccharides, SOD-like and nutrient compositions of water extracts. The content of total polysaccharides was obviously higher than flavonoids [49]. SVW, SVE60 and SVE95 were three extracts of *S. vaninii* which were separately extracted by water, 60% ethanol and 95% ethanol in this experiment. The SVW had the richest content in polysaccharides and lowest content in flavonoid compounds in those extracts. SVW60 had the highest flavonoid content, and its polysaccharide content was greater than that in SVE95. Therefore, the composition of SVE60 was analysed via UPLC-MS. The results suggested that the essential components were phenolics, and the majority were phenolic acids, flavonoids and phenylpropanoids. Many phenolic compounds have been shown to inhibit proliferation and angiogenesis of tumour cells in vitro [50].

The 95% ethanolic extract from the fruiting body of Sanghuang inhibited the proliferation of both cell lines in a dose-dependent manner, and the IC_50_ values at 48 h were 72 and 103 g/mL for SK-Hep-1 cells and RHE cells [51]. According to the CCK-8 cell proliferation results, the three extracts significantly reduced the cell survival rate with a good dose–effect relationship, and the effect of the two ethanol extracts was better than the water extract, especially SVE60, which had a much higher IC_50_ than SVW and SVW95. It is well known that apoptosis is an evolutionarily suicide program regulated by many genes that activates the cell death process, resulting in the removal of damaged tissue [52]. Furthermore, the two ethanol extracts induced SW480 apoptosis, particularly SVE60. SVE60 also had the ability to inhibit cancer cell proliferation by causing cell arrest in different phases.

Actually, while obvious inhibitory trends for cancer cells were observed in all dosages, there was very little apoptosis at high concentrations in this experiment, survival rates were low in all concentrations, and similar results were also found in cell cycle. They were probably caused by apoptosis or the death of cancer cells, due to the short detection time in these tests. SVE60 could significantly decrease the expression of mTOR, AKT and p-AKT proteins, which was related to the proliferation of SW480 cells and the activation of mTOR signalling. This study analysed the extract components of *S. vaninii* and explored its anti-colon cancer mechanism. These results showed that SVE60 had good potential for usage as an adjuvant chemotherapeutic and anti-colon cancer drug, in postoperative nutrition and in the prevention of recurrence.

The study demonstrated SVE60 had an obvious effect on anti-colon cancer. In a further study, we plan to prolong the determining time in apoptosis and cell cycle experiments so a clearer trend can be observed. Furthermore, we need to isolate and purify components from the 60% ethanol extract and observe the inhibition effect of the extract on human colon cancer in vivo via an orthotopic model of colon cancer in nude mice.

## Figures and Tables

**Table 1:** The MS data and the identification results of SVE60.

Peak No.	tR (min)	Identification	Formula	Mass (m/z)	Cacl. Mass (m/z)	mDa	Fragments
1	0.50	2-Carboxylbenzaldehyde	C_8_H_6_O_3_	151.0358	151.0395	−3.7	151,128,110
2	0.55	8-hydroxyl-5-O-β-D-Glucopyranosylpsoralen	C_17_H_16_O_10_	381.0803	381.0822	−1.9	381,365,353,258,104
3	0.55	7-(α-D-Glucopyranosyloxy)-2,3,4,5,6-pentahydroxyheptanoic acid	C_13_H_24_O_13_	387.1141	387.1139	0.2	387,341,245,181,129
4	0.62	2,3,4,5-Tetra-O-acetylhexonic acid	C_14_H_20_O_11_	365.1058	365.1084	−2.6	365,229,205,175,124
5	0.75	Adenosine	C_10_H_13_O_4_N_5_	268.1047	268.1046	0.1	268,245,229,136,124
6	0.75	Citric acid	C_6_H_8_O_7_	191.0190	191.0192	−0.2	191,173,128,111
7	1.34	Protocatechuic acid	C_7_H_6_O_4_	153.0183	153.0188	−0.5	153,109
8	2.01	Protocatechuic aldehyde	C_7_H_6_O_3_	137.0235	137.0239	−0.4	137,136
9	2.57	Caffeic acid	C_9_H_8_O_4_	179.0342	179.0344	−0.2	179,151,135,113
10	2.99	Ethyl 6-hydroxy-1-cyclohexene-1-carboxylate	C_9_H_14_O_3_	171.0999	171.1021	−2.2	229,171,158,138
11	3.78	2-(2-{2-[2-(2-Methoxyphenoxy) ethoxy]ethoxy} Ethoxy)ethanol	C_15_H_24_O_6_	299.1491	299.1495	−0.4	299,249,207,147,113
12	4.18	Osmundacetone	C_10_H_10_O_3_	177.0551	177.0552	−0.1	177,161,133
13	4.89	Hispidin	C_13_H_10_O_5_	245.0449	245.045	−0.1	245,159,113
14	5.33	Sternbin	C_16_H_14_O_6_	301.0706	301.0712	−0.6	301,257,249,179,113
15	5.55	2,6-bis[3-(3-tert-butyl-2-hydroxy-5-methylphenyl)-3-tricyclo[5.2.1.02,6]decanyl]-4-methylphenol	C_49_H_64_O_3_	701.4937	701.4934	0.3	701,680,340,229,138
16	5.80	Phelligridimer A or isomer	C_52_H_32_O_20_	977.1552	977.1565	−1.3	977,301,245,229,142
17	5.86	4,4′-[2,7-Naphthalenediylbis(oxy)] diphthalic acid	C_26_H_16_O_10_	487.0648	487.0665	−1.7	487,463,259,181,113
18	6.22	Davallialactone	C_25_H_20_O_9_	463.1021	463.1029	−0.8	463,379,259,159,113
19	6.29	Phelligridimer A or isomer	C_52_H_32_O_20_	977.1552	977.1565	−1.3	977,301,245,229,142
20	6.88	4-dimethyl methoxyphenylmethylene malonate	C_13_H_14_O_5_	249.0758	249.0763	−0.5	249,219,159,113
21	7.00	Unknown	C_49_H_78_O_18_	955.5246	955.5266	−2.0	956,423,301,229,149
22	7.12	Hosenkoside C	C_48_H_82_O_20_	977.5359	977.5321	3.8	978,932,113
23	7.89	Hypholomine B	C_26_H_18_O_10_	491.0981	491.0978	0.3	491.301,183,142
	8.01	Hypholomine B	C_26_H_18_O_10_	489.0830	489.0822	0.8	489,445,199,147,113
24	8.13	Acetyl-SSa	C_44_H_70_O_14_	823.4819	823.4844	−2.5	823,423,203,147,138
25	8.24	12-O-Acetylpergularin3-O-[β-D-oleandropyranosyl-(1→4)-β-D-canaropyranosyl-(1→4)-β-D-cymaropyranosyl-(1→4)-β-D-cymaropyranoside]	C_50_H_80_O_18_	969.5384	969.5423	−3.9	970,423,301,229,149
26	8.30	Unknown	C_36_H_78_O_21_	845.4923	845.4957	−3.4	845,445,249,130,113
27	8.43	Muricatin II	C_49_H_84_O_20_	991.5508	991.5478	3.0	992,946,113
28	10.43	Inoseavin A	C_25_H_18_O_9_	461.0863	461.0873	−1.0	461,377,159,135,113
29	14.01	Acetyl-SSa	C_44_H_70_O_14_	823.4802	823.4844	−4.2	823,801,301,229,142
30	14.15	Unknown	C_36_H_78_O_21_	845.4937	845.4957	−2.0	845,799,113
31	14.99	(3β,16β,24S)-cycloartane-3,16,24,25,30-pentol 3,25-di-β-D-glucopyranoside	C_42_H_72_O_15_	815.4830	815.4793	3.7	815,363,249,175,113
32	16.37	Unknown	C_61_H_66_O_2_	829.4944	829.4985	−4.1	829,786,385,147,113
33	17.26	5′,8′-dihydroxy-5,8-dimethoxy-6,6′-dimethyl-7,3′-binaphthyl- 1,4,1′,4′-tetraone	C_24_H_18_O_8_	433.0908	433.0923	−1.5	433,385,179,147,113
34	17.36	Unknown	C_56_H_90_O_23_	1131.5933	1131.5951	−1.8	1,131,407,229,138
35	17.48	Unknown	C_48_H_98_O_30_	1153.6039	1153.6065	−2.6	1,154,599,489,113
36	17.79	Chakasaponin VI	C_59_H_92_O_26_	1217.5917	1217.5955	−3.8	1,218,301,229,138
37	18.14	Unknown	C_41_H_80_O_24_	955.4695	955.4961	0.4	955,500,334,207,113
38	20.11	12-O-Acetyllineolon3-O-[β-D-oleandropyranosyl-(1→4)-β-D-digitoxopyranosyl-(1→4)-β-D-cymaropyranosyl-(1→4)-β-D-cymaropyranoside]	C_50_H_80_O_18_	969.5378	969.5423	−4.1	970,767,425,229,149
39	20.20	Muricatin IV	C_49_H_84_O_20_	991.5518	991.5478	4.0	992,946,113
40	20.42	Cladoloside A4	C_53_H_82_O_21_	1055.5393	1055.5427	−3.4	1,056,875,301,229
41	20.50	Unknown	C_44_H_88_O_26_	1031.5485	1031.5486	−0.1	1,032,992,207,113
42	21.20	3β-O-[β-D-glucopyranosyl-(1→2)-β-D-glucopyranosyl]-olean-12-en-28-O-[(3-O-acetyl)-α-L-rhamnopyranosyl] ester	C_50_H_80_O_18_	969.5368	969.5364	0.4	970,407,301,229,138
43	21.29	Merremoside c	C_49_H_84_O_20_	991.5511	991.5478	3.3	992,946,179,113

**Table 2:** The result of apoptosis induced by the ethanol extracts of *S. vaninii*.

Apoptosis Rate	SVE60				SVE95			
	Control	3 μg/mL	30 μg/mL	300 μg/mL	Control	3 μg/mL	30 μg/mL	300 μg/mL
Total	0.8%	2.8%	3.0%	8.3%	0.9%	1.9%	2.6%	8.2%
Early	0.7%	2.3%	2.7%	7.7%	0.8%	2.3%	2.7%	7.7%
Late	0.1%	0.5%	0.3%	0.6%	0.1%	0.3%	0.4%	0.6%

**Table 3:** Primer sequences used for the qRT-PCR.

Name	Forward Primer (5′→3′)	Reverse Primer (5′→3′)
AKT	ATGAACGACGTAGCCATTGTG	TTGTAGCCAATAAAGGTGCCAT
mTOR	ACCGGCACACATTTGAAGAAG	CTCGTTGAGGATCAGCAAGG

## Data Availability

The study did not report any data.
